# Supplementary material for: Unified single-cell analysis of testis gene regulation and pathology in five mouse strains
Source: eLife. 2019 Jun 25;8:e43966. doi: 10.7554/eLife.43966 (PMC6615865; doi:10.7554/eLife.43966)
Supplement: Supplementary file 3. — A table of key genes from 26 example components. [file elife-43966-supp3.docx]

Supplementary File 3: Key genes from example components of different stages

| **Stage (Component[s])** | **Seurat Cluster[s]** | **Example Key Genes** |
| --- | --- | --- |
| Undifferentiated Spermatogonia (50 & 31) | 6 | Negative C50 loadings:  Gfra1, Ccnd2, Glis3, Zfp462, Tex19.1, Dppa4  C50 loadings close to 0:  Zbtb16 aka Plzf, Sox4, Afp, Mageb4, Foxo1  Positive C50 loadings:  Nanos3, Lin28a, Foxf1, Pramef12, Sox3  (All have positive C31 loadings) |
| Differentiating Spermatogonia (7) | 6 | Glis2, Nanos1, Rcor2, Zswim5 |
| Spermatogonia (Broad) (33) | 6 | Uchl1, Dmrt1, Sohlh1, Dnmt3a, Dnmt3b, Dnmt1, Scml2, Msh2, Map7d2, Ung |
| Intermediate/B Spermatogonia (2) | 31 | Ctcfl, Esx1, Pou4f1 aka Brn-3a, Tex13b |
| (Pre)Leptotene (5) | 32 | DSB associated:  Prdm9, Setdb1, Dmc1, Gm960 (aka Top6bl), Brca2, Tex15, Ddb2, Brip1, Msh5, Mms22l, Meilb2 (Hsf2bp), Mcm8, Rad51, Ccdc36 (Iho1)  ZMM:  Shoc1 (AI481877), Msh5, Brip1  Cohesin & synaptonemal components:  Rad21l, Smc1b, Smc3, 4930447C04Rik (aka Six6os1), Tex12  Ctcfl regulated:  Prss50, Stra8, Ugt8a, Gal3st1  Telomere tethering:  Terb1, Terb2 |
| Zygotene (44) | 32 | Rad51ap2, Meiob, Spata22, Hfm1 |
| Early Pachytene (13) | 24, 25, 26 | Meiotic cell cycle: Ccna1, Ccnb3, Aurka, Plk1  piRNA associated  [Better known drosophila homologues in square brackets]:  Piwil1 [Miwi], Tdrd1 [Tudor], Tdrd5 [Tejas], Pld6 [Zucchini]  Protein folding: Hspa5, Calr, Hsp90b  Fertilization: Zpbp, Zpbp2, |
| Early Pachytene 2 (47) | 24, 25, 26 | Chromosome function: Hormad1, Setx, Ncaph,  Kdm1b  Spindle function: Cenpe, Cntrob, Pcm1  Meiotic cell cycle: Stambp, Ccnb1ip1, Ccnb3, Gfra4 |
| Mid Pachytene (48) | 7, 24, 25 | Cilium/axoneme assembly: Cfap46, Cfap65, Cfap74,  Dnah2, Dnah12, Dnah14, Dnhd1, Ak7, Ccdc39, Mroh2a  Microtubule/spindle function: Dcdc2b, Ccdc88a, Knl1  Splicing: Srrm2, Tra2a, Srek1, Rbm5, Rbm25 |
| Pachytene and late pachytene (42, 39) | 22, 24, 26 | piRNA associated [Better known drosophila homologues in square brackets]:  Piwil1 [Miwi], Tdrd1 [Tudor], Tdrd5 [Tejas], Tdrd12 [Yb], Piwil2 [Mili], Mael [Maelstrom], Pld6 [Zucchini], Exd1 [Egalitarian], Ddx4 aka Mvh [Vasa], Tdrkh [Papi], Tdrd9 [SpnE]  Meiotic cell cycle: Calm1, Calm2, Calm3, Meig1, Lyar, Spata4, Cetn1, Mns1  Translational Repression:  Ybx1 (aka MSY2), Ybx3, Pabpc1  Cilium assembly:  Rsph1, Ropn1l, Dnah8, Dnaaf1, Cfap36, Bbof1, Ccdc39  Post-meiotic (fertilization and metabolism):  Ldhc, Dkkl, Clgn, Spink2, Catsperz, Fbp, Cct1, Cct3, Cct4, Cct7 |
| Diplotene & Divisions (20) | 22 | Cell cycle:  Ccnb1, Ccna1, Cdc25a, Aurka, Bora, Plk1, Rgcc, Fzr1  DUF622 containing:  1700001F09Rik, Gm3453, Gm10354, Gm3149, Gm8362, Gm3127, Gm17019, Gm4181, Speer4e, Speer4b, Gm9758, Gm8232, BC061237, Gm5458, 4930572O03Rik, Gm5800, Gm7361, Gm8220  SSXRD and KRAB-related domain containing:  Ssxb1, Ssxb2, Ssxb3, Ssxb5, Ssxb6  Others:  Tbpl1 (aka Tlf) |
| Spermatid - Acrosome (30) | 17, 19 | Acrosomal:  Spaca1, Spaca3 (aka Lyzl3), Spaca4, Spaca5 (aka Lyzl5), Spaca7; Lyzl1, Lyzl4, Lyzl4os, Lyzl6; Acrv1, Aep1, Spata9, Spata31, & Spata46  Sperm-oocyte interaction:  Izumo1, Izumo3, Zpbp, Zp3r  Others:  Catsper1, Catsper3, & Catsper4;  Tekt1, Tekt2, Tekt3, & Tekt4;  Creb3l4 aka Atce1, and 1700016D06Rik + Lrcc34 |
| Spermatid - Mysterious (35) | 17, 10 | Testis enriched genes of unknown function: Tex29, Lrrd1, Smco4, Heatr9, Hsfy2, Tepp, Spata31d1d, Tmem81, Spata25  Mitochondrial function: Crls1, Slc25a41 |
| Spermiogenesis (17 [& 18, 34]) | 13, 20, 21 | Histone Replacements:  Prm1, Prm2, Prm3, Tnp1, Tnp2  Others:  Smcp, Odf2, Gapdhs, Oaz3, H1fnt (aka H1t2), Pgk2, and Cabs1 4+ Abhd5 |
| Leydig (40) | 4, 5 | Testosterone Biosynthesis:  Star, Cyp11a1, Hsd3b1, Cyp17a1, Hsd17b3  Others:  Insl3, Ptgds |
| Sertoli (45) | 8, 3 | Aard, Defb36, Cst12, Ldhb, Tmsb4x, Cst9, Gstm6, Sin3b, Gsta4, Chchd10, Gstm7, Basp1, Wfdc10 |
| Macrophages (11) | 1 | Csf1r, Cd163, Cd68, Adgre1 (aka F4/80), Itgam (aka CD11b), Mrc1, Cx3cr1, Fcgr3, C1qa, C1qb, C1qc |
| T-cells (3) | 1 | Ptprc (aka CD45), Il2rg, Cd3g, Cd3d, Cd3e, Trbc2, Trac, Ms4a4b, and Cd2 |
| Telocyte (32) | 2 | Dcn, Cd34, Pdgfra, Col1a2, Col3a1, Col6a1, Col4a4, Col4a1, Col1a1, Lamb1, Lama2, Lamb2, Des |
| Peritubular Myoid (21) | 3 | Cnn3, Vegfa, Edn1 |
| Hormad1 KO (38) | 30 | X & Y linked:  Zfy1, Zfy2, Rhox2h, Rhox2d, Rhox2a, Rhox2c, Rhox2g  Autosomal:  Dnajc12, A830018L16Rik (aka C8orf34) |
| Cul4a KO (25) | 17, 10 | Hist1h2al, Csmd1, Jakmip2, Tagln2, Map2k7, Lpo |
| Respiration (9) | 22, 23, 26, 27 | Complex I (NADH:ubiquinone oxidoreductase)  Ndufa11, Ndufa12, Ndufa2, Ndufa3, Ndufa5, Ndufa6, Ndufa7, Ndufaf2, Ndufaf8, Ndufb10, Ndufb2, Ndufb4, Ndufb5, Ndufb7, Ndufb9, Ndufc1, Ndufs4, Ndufs6, Ndufv3  Complex III (Ubiquinol-cytochrome c reductase)  Uqcc2, Uqcr10, Uqcr11, Uqcrb, Uqcrh, Uqcrq  Complex IV (Cytochrome c oxidase) subunits  Cox17, Cox4i1, Cox5a, Cox5b, Cox6a1, Cox6b2, Cox6c, Cox7a2, Cox7b2, Cox7c, Cox8c  Cytochrome c:  Cyct  Complex V (ATP Synthase)  Atp5e, Atp5h, Atp5j, Atp5j2, Atp5k, Atpif1 |
| Batch Effect (22) | 17, 19, 28 | (all downregulated)  Ribosomal Proteins:  Rps7, Rpl11, Rps13, Rps12, Rps17, Rps23, Rpl18a, Fau (Rps30 fusion)  Others:  Tpt1, Kpna2, Eif1 |
| Batch Effect (12) | 31, 6 | Gm42418, Rbm25, mt-Rnr1, mt-Rnr2, Ncl, Pet2, Vps8 |
