# Supplementary material for: Unified single-cell analysis of testis gene regulation and pathology in five mouse strains
Source: eLife. 2019 Jun 25;8:e43966. doi: 10.7554/eLife.43966 (PMC6615865; doi:10.7554/eLife.43966)
Supplement: Supplementary file 6. [file elife-43966-supp6.docx]

| Component | GO Category | Genes | Adjusted p-value |
| --- | --- | --- | --- |
| 49N (Sertoli/Leydig) | GO:0042982  amyloid precursor protein metabolic process | Clu/Itm2b/App/Tmed10/Psen2/Apoe/Itm2c | 0.00072 |
| 26N (Leydig) | GO:0097242  amyloid-beta clearance | Clu/Ldlr/Igf1r/C3 | 0.00771 |
| 16N (rare Sertoli-like) | GO:1904645  response to amyloid-beta | Casp4/Lrp1/Igf1r/Fyn/Cacna2d1 | 0.00974 |

Supplementary File S6. GO Categories related to amyloid-beta metabolism show significant enrichment in components 49, 26 and 16. For each component we provide the component description, the GO category description, the genes from that category that were detected as a part of that component, as well as the multiple-test-corrected p-value corresponding to a test for enrichment.
